# Identification of metabolism-related subtypes and feature genes in Alzheimer’s disease

**DOI:** 10.1186/s12967-023-04324-y

**Published:** 2023-09-15

**Authors:** Piaopiao Lian, Xing Cai, Cailin Wang, Ke Liu, Xiaoman Yang, Yi Wu, Zhaoyuan Zhang, Zhuoran Ma, Xuebing Cao, Yan Xu

**Affiliations:** 1 Department of Neurology, Union Hospital, Tongji Medical College, Huazhong University of Science and Technology, Wuhan, China; 2 Department of Oncology, Union Hospital, Tongji Medical College, Huazhong University of Science and Technology, Wuhan, China

**Keywords:** Alzheimer’s disease, Metabolic subclass, Immune infiltration, Characteristic genes, Machine learning

## Abstract

**Background:**

Owing to the heterogeneity of Alzheimer's disease (AD), its pathogenic mechanisms are yet to be fully elucidated. Evidence suggests an important role of metabolism in the pathophysiology of AD. Herein, we identified the metabolism-related AD subtypes and feature genes.

**Methods:**

The AD datasets were obtained from the Gene Expression Omnibus database and the metabolism-relevant genes were downloaded from a previously published compilation. Consensus clustering was performed to identify the AD subclasses. The clinical characteristics, correlations with metabolic signatures, and immune infiltration of the AD subclasses were evaluated. Feature genes were screened using weighted correlation network analysis (WGCNA) and processed via Gene Ontology and Kyoto Encyclopedia of Genes and Genomes pathway analyses. Furthermore, three machine-learning algorithms were used to narrow down the selection of the feature genes. Finally, we identified the diagnostic value and expression of the feature genes using the AD dataset and quantitative reverse-transcription polymerase chain reaction (qRT-PCR) analysis.

**Results:**

Three AD subclasses were identified, namely Metabolism Correlated (MC) A (MCA), MCB, and MCC subclasses. MCA contained signatures associated with high AD progression and may represent a high-risk subclass compared with the other two subclasses. MCA exhibited a high expression of genes related to glycolysis, fructose, and galactose metabolism, whereas genes associated with the citrate cycle and pyruvate metabolism were downregulated and associated with high immune infiltration. Conversely, MCB was associated with citrate cycle genes and exhibited elevated expression of immune checkpoint genes. Using WGCNA, 101 metabolic genes were identified to exhibit the strongest association with poor AD progression. Finally, the application of machine-learning algorithms enabled us to successfully identify eight feature genes, which were employed to develop a nomogram model that could bring distinct clinical benefits for patients with AD. As indicated by the AD datasets and qRT-PCR analysis, these genes were intimately associated with AD progression.

**Conclusion:**

Metabolic dysfunction is associated with AD. Hypothetical molecular subclasses of AD based on metabolic genes may provide new insights for developing individualized therapy for AD. The feature genes highly correlated with AD progression included *GFAP*, *CYB5R3*, *DARS*, *KIAA0513*, *EZR*, *KCNC1*, *COLEC12*, and *TST*.

**Supplementary Information:**

The online version contains supplementary material available at 10.1186/s12967-023-04324-y.

## Background

Alzheimer's disease (AD) is the most prevalent type of dementia and affect > 50 million individuals worldwide [1]. The primary pathological characteristics of AD are the buildup of amyloid-β (Aβ) plaque and intraneuronal neurofibrillary tangle (NFT) [2]. Aβ plaques occur owing to the successive enzymatic breakdown of amyloid precursor protein by β-secretase and γ-secretase [3]. Despite decades of research, the pathogenic mechanism of AD remains unclear and the current treatments are unsatisfactory let alone curative [4]. Therefore, early diagnosis and intervention are necessary for patients with AD. However, AD diagnosis has long been a challenge, and current biomarkers are inadequate to provide personalized genetic-level treatments. Thus, molecular subtypes may help identify the heterogeneity among patients with AD and facilitate the discovery of targeted therapies for AD.

Mounting evidence suggests that AD is a wide-ranging metabolic disorder characterized by disrupted glycolipid and energy metabolism. These metabolic abnormalities may contribute to the severity of AD neuropathology and the eventual manifestation of AD symptoms [5–9], thus emphasizing the crucial role of metabolism in AD and elevating the prominence metabolism dysfunction in AD research. Therefore, it is necessary to explore the metabolism-related subtypes and feature genes of AD.

In this study, we integrated eight AD datasets, including 737 patients with AD, into a single dataset for clustering analysis based on metabolic genes. Through consensus clustering, we identified three distinct subclasses of AD, which were designated as Metabolism Correlated (MC) A (MCA), MCB, and MCC subclasses. Subsequently, we evaluated the clinical characteristics, correlations with metabolic signatures, immune infiltration patterns, and prognostic implications of these AD subclasses. Weighted correlation network analysis (WGCNA) R package was employed to identify the module most associated with poor AD progression, and we performed a functional enrichment analysis of the genes associated with this module. To further narrow down the selection of the feature genes, three machine-learning algorithms were employed, including Support Vector Machines (SVM), least absolute shrinkage and selection operator (LASSO) regression, and Random Forest (RF). Thus, we successfully identified eight core genes exhibiting outstanding diagnostic potential and serving as promising therapeutic targets for AD.

## Methods

### Data collection and processing

The gene expression data of patients with AD were obtained from the Gene Expression Omnibus (GEO) database (https://www.ncbi.nlm.nih.gov/geo/) [10]. The following eight datasets were selected: GSE48350, GSE5281, GSE28146, GSE122063, GSE118553, GSE8442201 (GSE84422 includes three subsets and GSE8442201 was annotated by GPL570), GSE132903, and GSE106241. A detailed description of these datasets is provided in Additional file 3: Table S1. We performed data filtering, background correction, log2 transformation, and normalization of these datasets. In addition, we merged the datasets and applied a batch correction using the Combat method from the "sva" package.

### Identification of AD subclasses

For consensus clustering [11], we utilized a previously published compilation of 2,752 metabolism-relevant genes [12], which encode all known human metabolic enzymes and transporters. Our aim was to classify the AD samples into distinct subclasses using consensus clustering. The maximum number of clusters was 5 and a filter was applied based on a cluster consensus score threshold of > 0.8.

### Gene set variation analysis

Gene set variation analysis (GSVA) represents an unsupervised and nonparametric approach to gene set enrichment analysis that estimates the score attributed to a particular pathway or signature based on transcriptomic data [13]. We acquired 84 metabolism-relevant gene signatures from previously published study [12]. By utilizing the GSVA R package, we calculated 120 scores for each sample corresponding to these 84 metabolism signatures.

### Evaluation of immune infiltration

Various algorithms are employed to assess the status of immune infiltration. The XCELL package was used to quantify the relative abundance of immune and stromal cells between the AD subclasses based on their gene expression profiles. The EPIC [14], ssGSEA [15], quanTIseq [16], TIMER [17], CIBERSORT [18], MCPCounter [19], XCELL [19], and ESTIMATE [20] algorithms were employed to calculate the ESTIMATE score and relative abundance of immune cells.

### Weighted correlation network analysis

The WGCNA package was used to establish a WGCNA network to identify gene modules associated with the three AD subclasses and the clinical characteristics of patients with AD [21]. To determine the optimal soft-threshold power, we employed a scale-free topology standard. Subsequently, we generated a weighted adjacency matrix and transformation of a topological overlap matrix. Hierarchical clustering and tree analysis was performed to screen modules containing > 50 genes. Each module was visually represented using an arbitrary color. The module eigengene represented each of the distinct modules. The traits examined in this study included the AD subclasses and several clinical features, such as NFTs and Braak.

### Functional enrichment analysis

R package “clusterProfiler” [22] was used to perform Gene Ontology (GO) and Kyoto Encyclopedia of Genes and Genomes (KEGG) analyses to identify the functions and pathways of hub genes in the cyan module.

### Machine learning

Stable and robust features play a crucial role in forecasting the onset and advancement of AD. We developed three machine-learning models: RF, LASSO regression, and SVM. The RF algorithm, known for its effectiveness and popularity, utilizes a majority voting approach to combine decision trees, resulting in high precision and rapid autonomous learning across diverse datasets. The LASSO regression algorithm, a well-established linear prediction method, makes predictions based on regression coefficients and has been extensively applied in various fields [23]. The SVM algorithm, a widely used machine-learning technique, projects input data into a higher-dimensional feature space by mapping a kernel function, thus facilitating classification compared with the original feature space [24]. Through an iterative learning process, SVM converges to the optimal hyperplane that maximizes interclass span. These machine-learning models were built based on an earlier study [25].

### Establishment and assessment of a nomogram

The combined dataset comprised 1262 samples, including 525 normal samples and 737 AD samples. These samples were randomly partitioned into testing (20%, N = 252) and training (80%, N = 1010) datasets. The feature genes were used to develop a nomogram using the “rms” package with the training set. The effectiveness of the nomogram was assessed separately for the test and training datasets. Calibration curves were employed to assess the predictive performance of the nomogram model. Finally, the clinical value of the model was assessed via decision curve analysis (DCA) and by examining the area under the curve (AUC) values.

### Assessment of the diagnostic significance of feature genes in AD

To assess the discriminative capacity of the feature genes for non-AD controls and patients with AD, we used eight datasets: GSE5281, GSE48350, GSE118553, GSE28146, GSE122063, GSE132903, GSE8442201, and GSE1297. The diagnostic performance of these feature genes was visualized by plotting the AUC using the R package of “pROC”.

### Animals

The P301S mouse, which carries the human tau gene with the P301S mutation, is a well-characterized mouse model used to study AD. P301S transgenic mice were a gift from Professor Gang Li at the Department of Neurology, Union Hospital, Tongji Medical College, Huazhong University of Science and Technology [26]. This transgenic mouse has a C57Bl/6 J background. All transgenic and nontransgenic mice were littermates of P301S mice. The 8-month-old P301S mice (male, n = 3) were used as an in vivo AD model and age-matched male C57BL/6 J mice (n = 3) were used as controls. The mice were housed under standard laboratory conditions and maintained in an artificial 12/12 h light/dark cycle. Food and water were provided ad libitum. All animal experiments were reviewed and approved by the Ethics Committee of Tongji Medical College, Huazhong University of Science and Technology.

### Quantitative reverse-transcription polymerase chain reaction

The cortices of the mice were surgically removed and stored at − 80 °C for subsequent biochemical analysis. The total RNA was extracted using TRIzol reagent. The mRNA was reverse transcribed to cDNA using a reverse transcription kit (Takara, Japan) according to the manufacturer's instructions. The cDNA, primers, and ChamQ SYBR qPCR Master Mix (Vazyme, China) were combined into a polymerase chain reaction (PCR) reaction plate and the mRNA levels of GFAP, CYB5R3, DARS, KIAA0513, EZR, KCNC1, COLEC12, and TST were measured using StepOnePlus real-time PCR System. All experiments were repeated thrice and the primer sequences are listed in Additional file 3: Table S2.

### Statistical analysis

Statistical analyses were conducted using R language (version 4.2.0). Between-group comparisons were conducted via Wilcoxon test. A P-value of < 0.05 was considered statistically significant.

## Results

### Consensus clustering identifies three AD subclasses

**Fig. 1:**
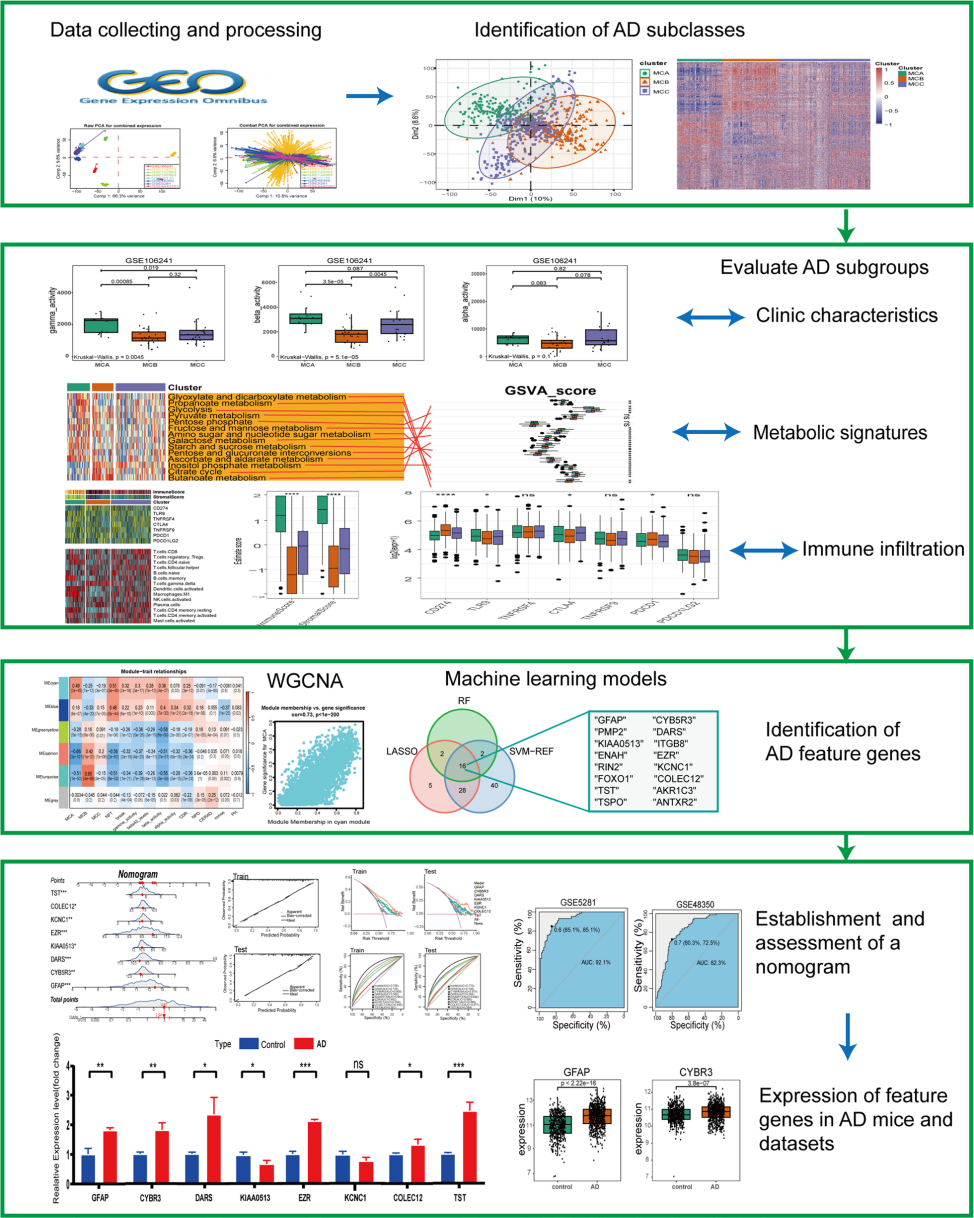
Flowchart of the research

**Fig. 2:**
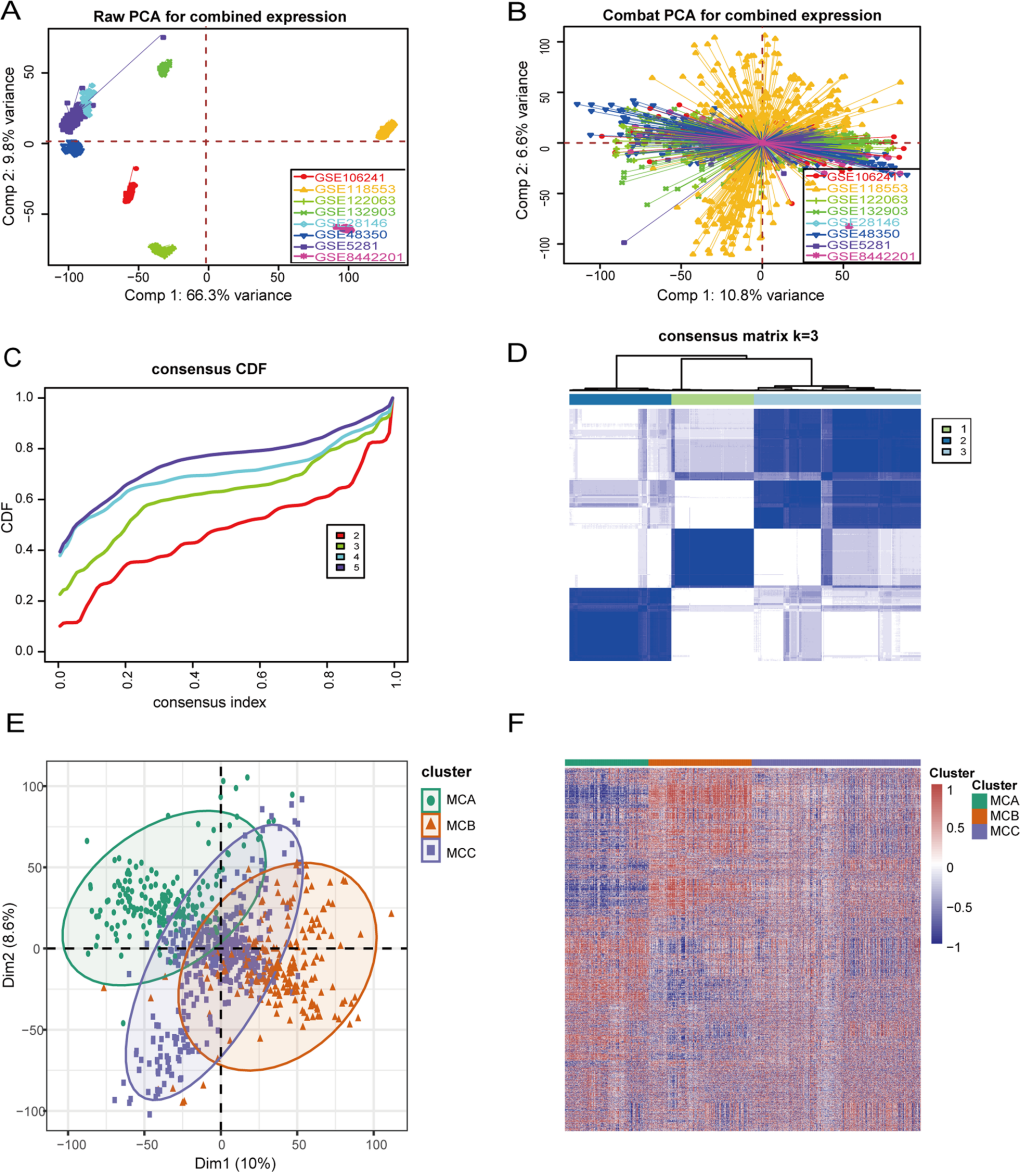
Identification of the AD subclasses. **A**, **B** Principal component analysis (PCA) of eight datasets before **A** and after **B** batch effect removal. **C** Consensus clustering utilizing metabolism-associated genes. CDF curve for k = 2–5 is shown. **D** Consensus clustering matrix when k = 3 **E** PCA supported stratification into three AD subclasses. **F** Metabolism-associated genes expression heatmap for the three AD subclasses. Red indicates high expression. Blue indicates low expression. White indicates no difference

The flowchart systematically describes our study (Fig. 1). Based on the previously reported 2,752 metabolism-related genes, consensus clustering classified the gene expression profiles for 743 AD samples after removing the batch effect (Fig. 2A, B) into distinct subclasses. They were categorized into two to five subclasses (Additional file 1: Fig. S1). After comprehensive consideration, k = 3 was determined as the optimal number of clusters. When k = 3, the CDF plot displayed the minimum fluctuation and the consensus matrix heatmap exhibited clear and distinct boundaries (Fig. 2C, D). Both principal component analysis (PCA) and a metabolism-associated genes expression heatmap unveiled significant discrepancies in the expression profiles between the three subclasses (Fig. 2E, F).

### Clinical characteristics of the AD subclasses

**Fig. 3:**
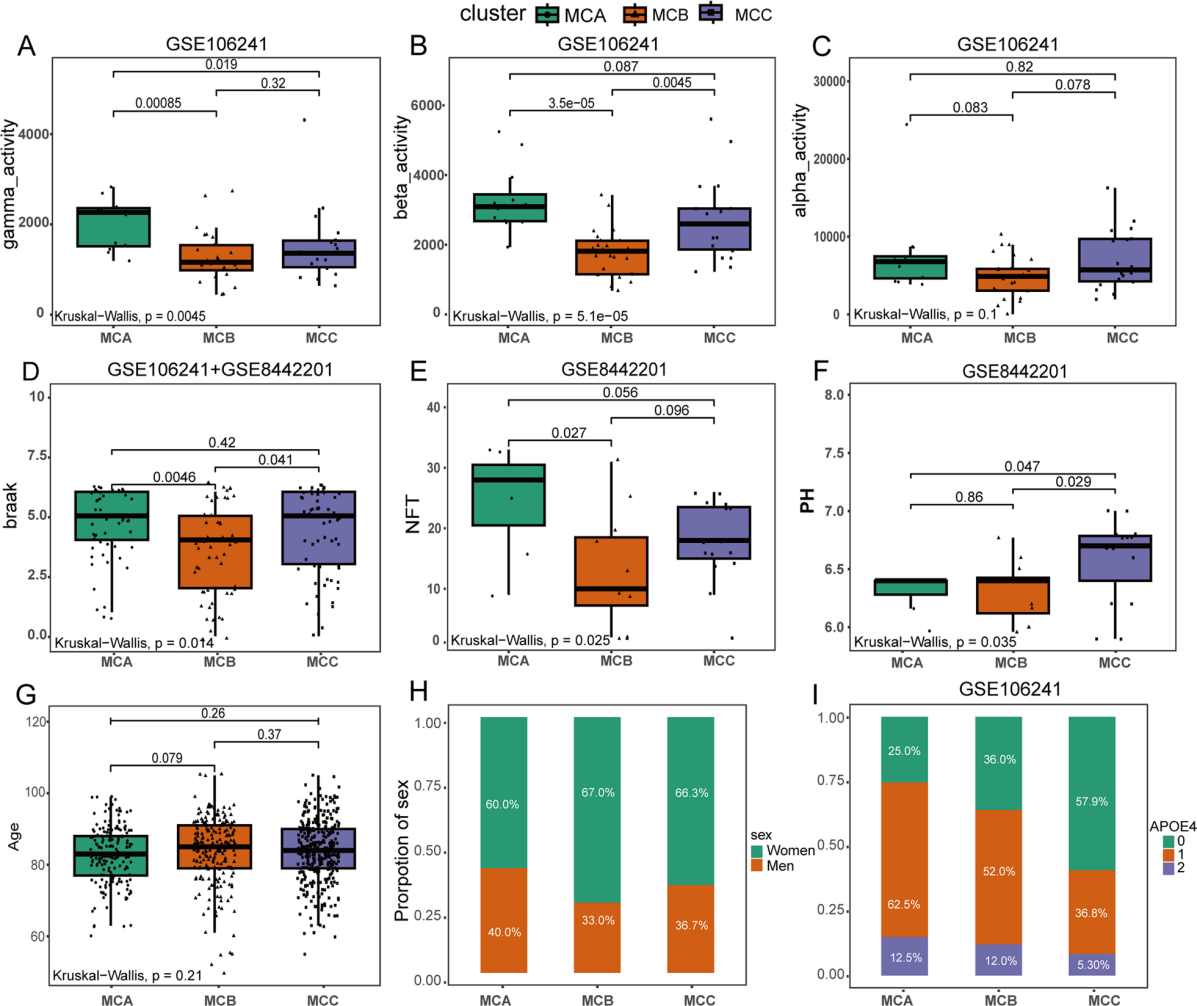
Clinical characteristics of the AD subclasses. **A** Comparison of gamma-secretase activity between each subclass. **B** Comparison of beta-secretase activity between each subclass. **C** Comparison of alpha-secretase activity between each subclass. **D** Comparison of Braak between each subclass. **E** Comparison of NFTs between each subclass. **F** Comparison of pH between each subclass. **G** Comparison of age between each subclass. **H** Proportion of sex between each subclass. **I** Proportion of APOE4 alleles in each subclass. (0 indicates no APOE4 allele, 1 represents one APOE4 allele, and 2 represents two APOE4 alleles). The clinical data of Fig. **A**–**C**, H is from GSE106241 (MCA, n = 16; MCB, n = 25; MCC, n = 19). The clinical data of Fig. **E**, **F** is from GSE8442201 (MCA, n = 7; MCB, n = 12; MCC, n = 15). The clinical data of Fig. **D** is from GSE106241 and GSE8442201 (MCA, n = 23; MCB, n = 37; MCC, n = 34). The clinical data of Fig. **G**, **I** are from eight merged datasets (MCA, n = 173; MCB, n = 274; MCC, n = 350). Kruskal–Wallis test was used to compare statistical differences

The gamma-secretase activity in MCA was notably higher compared with that in MCB and MCC (P < 0.001 or 0.05; Fig. 3A). Compared with MCB, beta-secretase activity, NFTs, and Braak were elevated in MCA and MCC (P < 0.01; Fig. 3B, D, E). The PH in MCC was higher compared with that in MCA and MCB (P < 0.05; Fig. 3F). The three AD subclasses contained a greater proportion of women than men (Fig. 3H). Furthermore, the proportion of one and two APOE 4 alleles was significantly higher in MCA compared with that in MCB and MCC (Fig. 3I). With respect to age and alpha-secretase activity, there was no difference between the three AD subclasses (Fig. 3C, G). The tissue origin of MCA, MCB, and MCC is shown in Additional file 2: Fig. S2.

### Association between the AD subclasses and metabolism-associated signatures

**Fig. 4:**
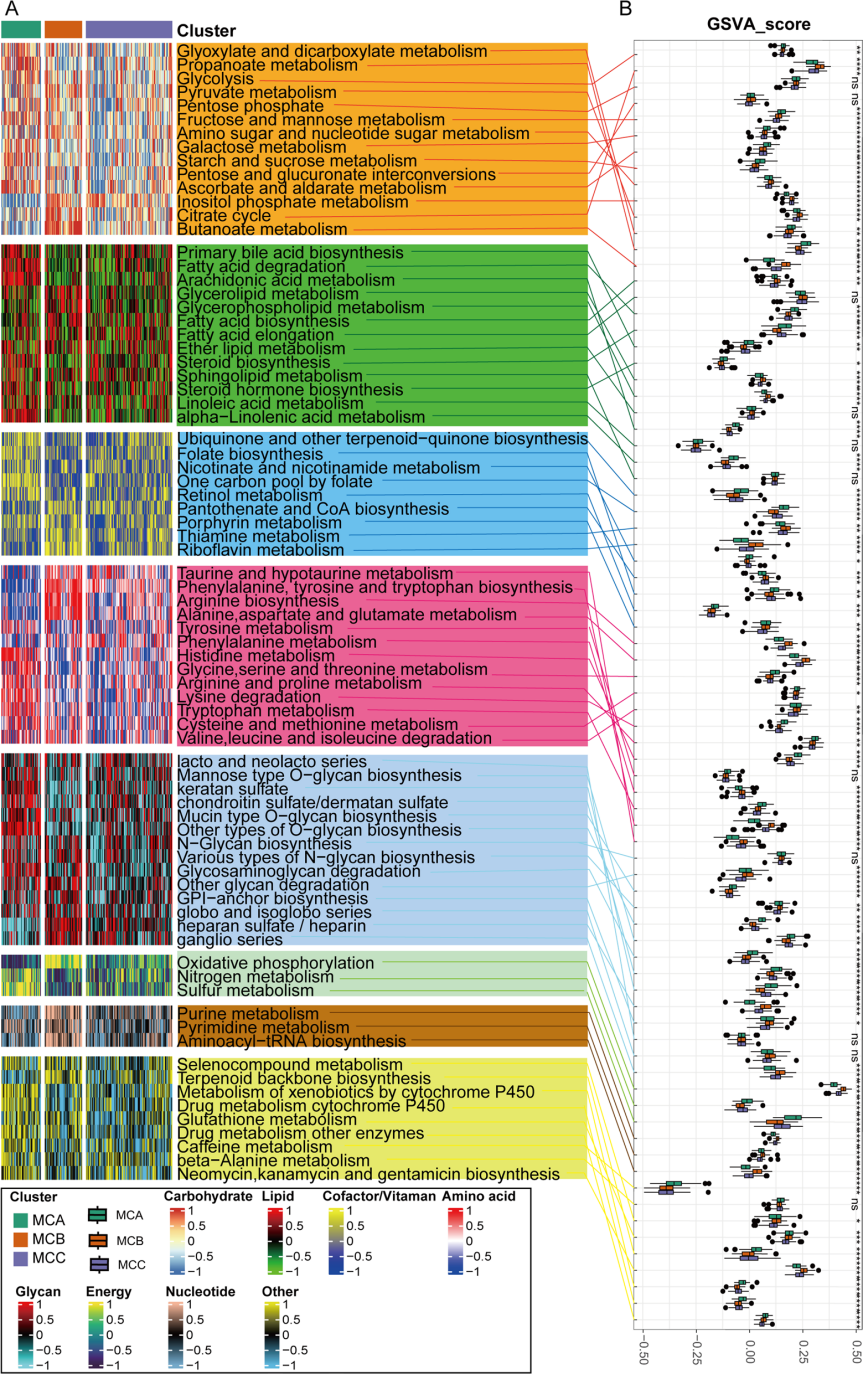
Association between metabolism-associated signatures and the AD subclasses. **A** Heatmap of metabolism-associated signatures. **B** Boxplot of the signature score for the metabolism-associated signatures distinguished by different subclasses (ns indicates no significance, *P < 0.05, **P < 0.01, ***P < 0.005, ****P < 0.0001)

Given that the AD subclasses were established based on metabolism genes, we investigated whether the different subclasses exhibited varying metabolic signatures. Initially, 84 metabolism processes were measured utilizing the “GSVA” R package. Next, we performed a differential analysis to identify the subclass-specific metabolic signatures, which were identified as signatures with a greater GSVA score in the relevant subclasses. The results indicated that only MCA and MCB exhibited distinct metabolism signatures of 40 and 30, respectively, whereas MCC exhibited negligible distinct metabolism signatures. Notably, 7 of the 40 distinct metabolism signatures in MCA were associated with carbohydrate metabolism (Fig. 4).

MCA was primarily associated with gene signatures for carbohydrate and lipid metabolism, with carbohydrate metabolism primarily comprising genes related to glycolysis, fructose, mannose, and galactose metabolism, whereas the genes related to citrate cycle and pyruvate metabolism were downregulated compared with the other two subclasses. Lipid metabolism in MCA mainly included fatty acid degradation. MCB was primarily associated with amino acid biosynthesis, nucleotides biosynthesis, and the citric acid cycle.

### Association between the AD subclasses and immune infiltration

**Fig. 5:**
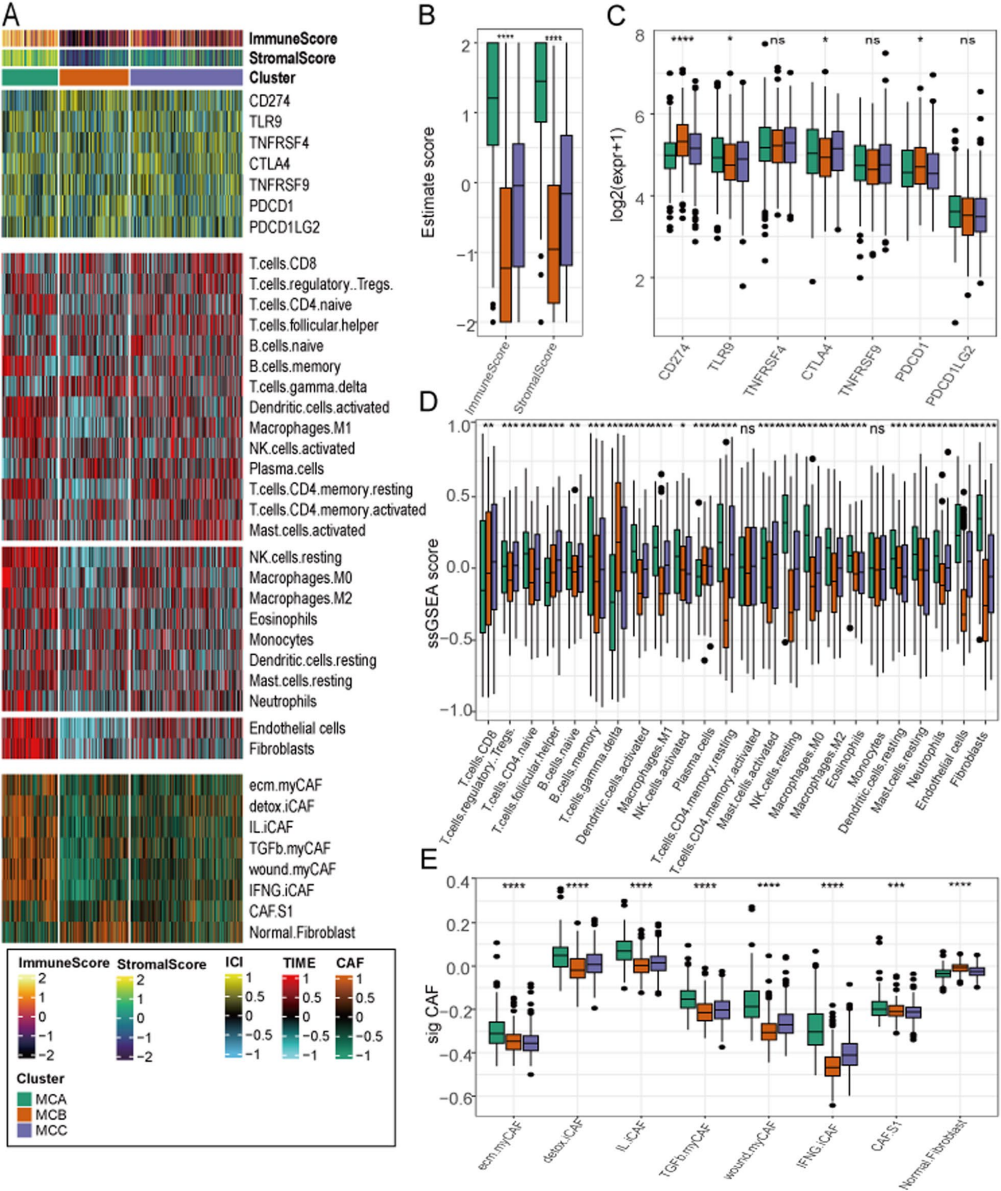
Association between AD subclasses and immune infiltration. **A** Heatmap describing the immune infiltration landscape in the three AD subclasses. Boxplots describing the distribution of expression for **B** the immune and stromal score, **B** immune checkpoint targets, **C** TME cells, and **D** fibroblast signatures in the three AD subclasses. (ns indicates no significance, *P < 0.05, **P < 0.01, ***P < 0.005, ****P < 0.0001)

To determine the characteristics of the AD subclasses, the ESTIMATE algorithm was applied to calculate the immune and stromal scores. The immune scores displayed a marked difference across the three groups, whereby MCA demonstrated a higher immune score compared with MCB and MCC (P < 0.0001; Fig. 5B). Furthermore, MCA exhibited a higher stromal score compared with those of MCB and MCC (P < 0.0001; Fig. 5B). Owing to the observed difference in the immune scores among the AD subclasses, immune infiltration was further examined to characterize the immunological landscape. We quantified the abundance of 24 microenvironment cell and analyzed the samples for the expression of immune checkpoints (Fig. 5A). Compared with other subclasses, we observed higher expression of several immune checkpoint genes in MCB, which may serve as targets for immunotherapy, including *CD274* (PDL1) and *PDCD1* (PDL2; Fig. 5C). In addition, MCA exhibited higher abundance of 18 immune cell populations (regulatory T cells, CD4 + T cells, nature B cells, memory B cells, activated dendritic cells, M1 macrophages, activated natural killer cells, memory CD4 + T cells, activated mast cells, resting natural killer cells, M0 macrophages, M2 macrophages, eosinophils, resting dendritic cells, resting mast cells, neutrophils, endothelial cells, and fibroblasts) compared with MCB or MCC (Fig. 5D). Notably, MCA demonstrated a higher infiltration of endothelial cells and fibroblasts (Fig. 5D). Therefore, we quantified the various types of cancer-associated fibroblasts (CAFs) and observed that MCA exhibited an enrichment of all the distinct subtypes of fibroblasts. Furthermore, MCA exhibited a depletion of normal fibroblasts (Fig. 5E).

### WGCNA to identify poor AD progression-associated module and hub genes

**Fig. 6:**
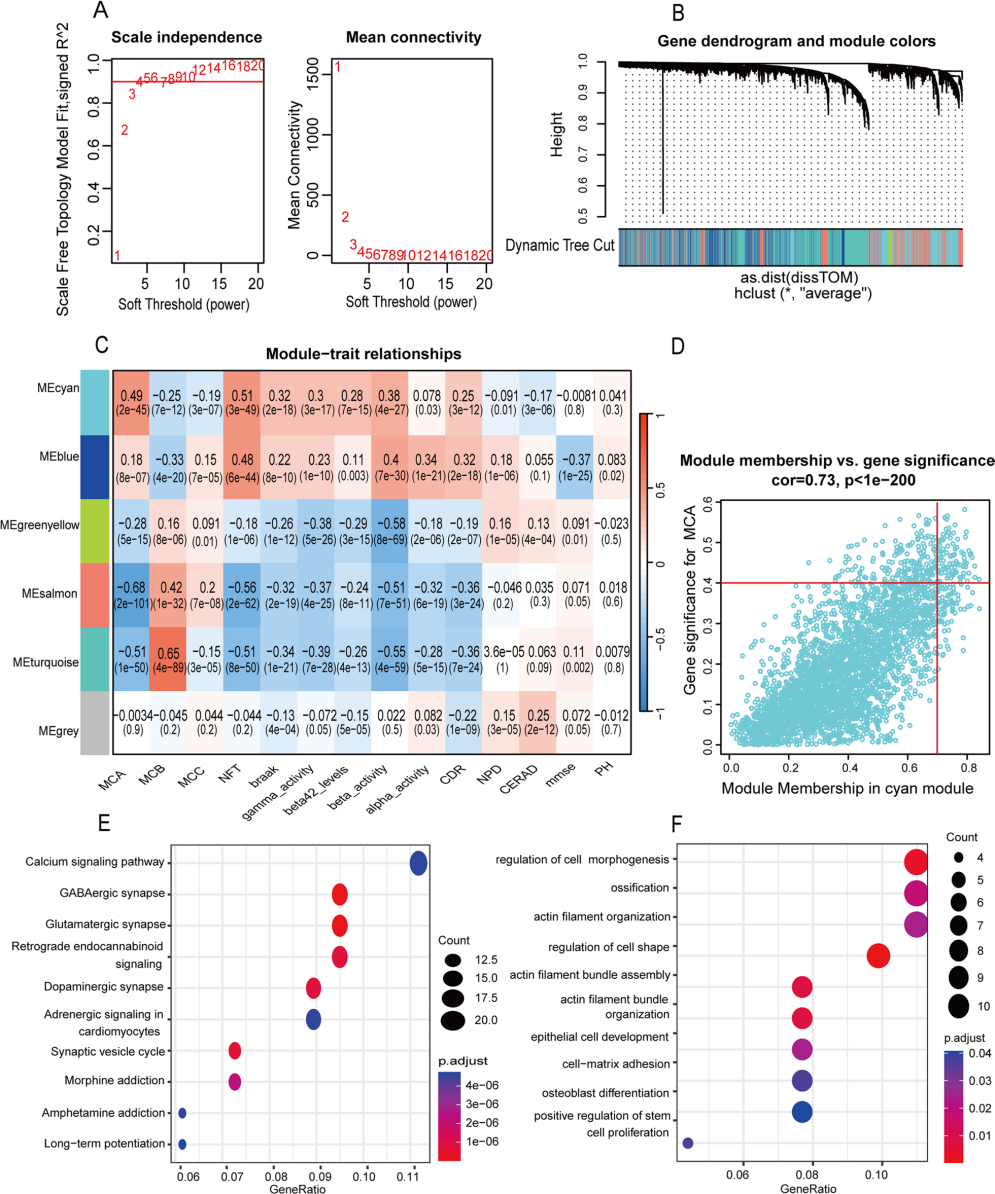
Identification of AD feature genes and functional enrichment analysis **A** Analysis of the scale-free index and the mean connectivity for various soft-threshold powers. **B** Identification of coexpression gene modules. The branches of the dendrogram clustered into six modules, each labeled with a unique color. **C** Heatmap showing the correlation between modules and feature gene sets. The framed cyan module was the key module most relevant to the MCA subclass. Gene significance and corresponding p values were calculated and are shown in the heatmap. **D** Scatter diagram showing the correlation of gene significance in cyan module with gene significance for MCA subclass. **E**, **F** Dot-plots showing the KEGG **E** and GO **F** enrichment analysis of hub genes

We conducted WGCNA using the merged dataset to identify the module associated with poor AD progression. When the soft-threshold was 4, the scale-free network and connectivity exhibited maximum efficiency (Fig. 6A). Using a hierarchical clustering algorithm, the clustering tree was classified into six gene modules, each of which assigned a unique color (Fig. 6B). Of these, the cyan module comprised 3284 genes and exhibited the strongest positive correlation with MCA (R = 0.49) as well as a series of AD-related high-risk indicators, including NFTs (R = 0.51), Braak (R = 0.32), gamma-secretase activity (R = 0.3), amyloid-beta 42 (R = 0.28), and alpha-secretase activity (R = 0.25) (Fig. 6C). Therefore, the cyan module was chosen as the hub module from which hub genes were extracted using the selection criteria cor.MM > 0.7 and cor.GS > 0.4 (Fig. 6D). In addition, we performed GO and KEGG enrichment analyses using the aforementioned hub genes (Fig. 6E, F). KEGG enrichment analysis revealed that various synapses, including GABAergic, glutamatergic, and dopaminergic synapses as well as synaptic transmission–related signaling pathways, including the calcium, adrenergic, and synaptic vesicle cycle signaling pathways, were closely associated with these hub genes (Fig. 6E, Additional file 3: Table S3). GO enrichment analysis revealed that these hub genes were predominantly enriched in cell morphogenesis regulation, actin filament organization, actin filament bundle assembly, actin filament bundle organization, and cell–matrix adhesion (Fig. 6F, Additional file 3: Table S4). These results indicate the important functions of these genes.

### Selection of the AD feature genes based on hub genes of cyan module

**Fig. 7:**
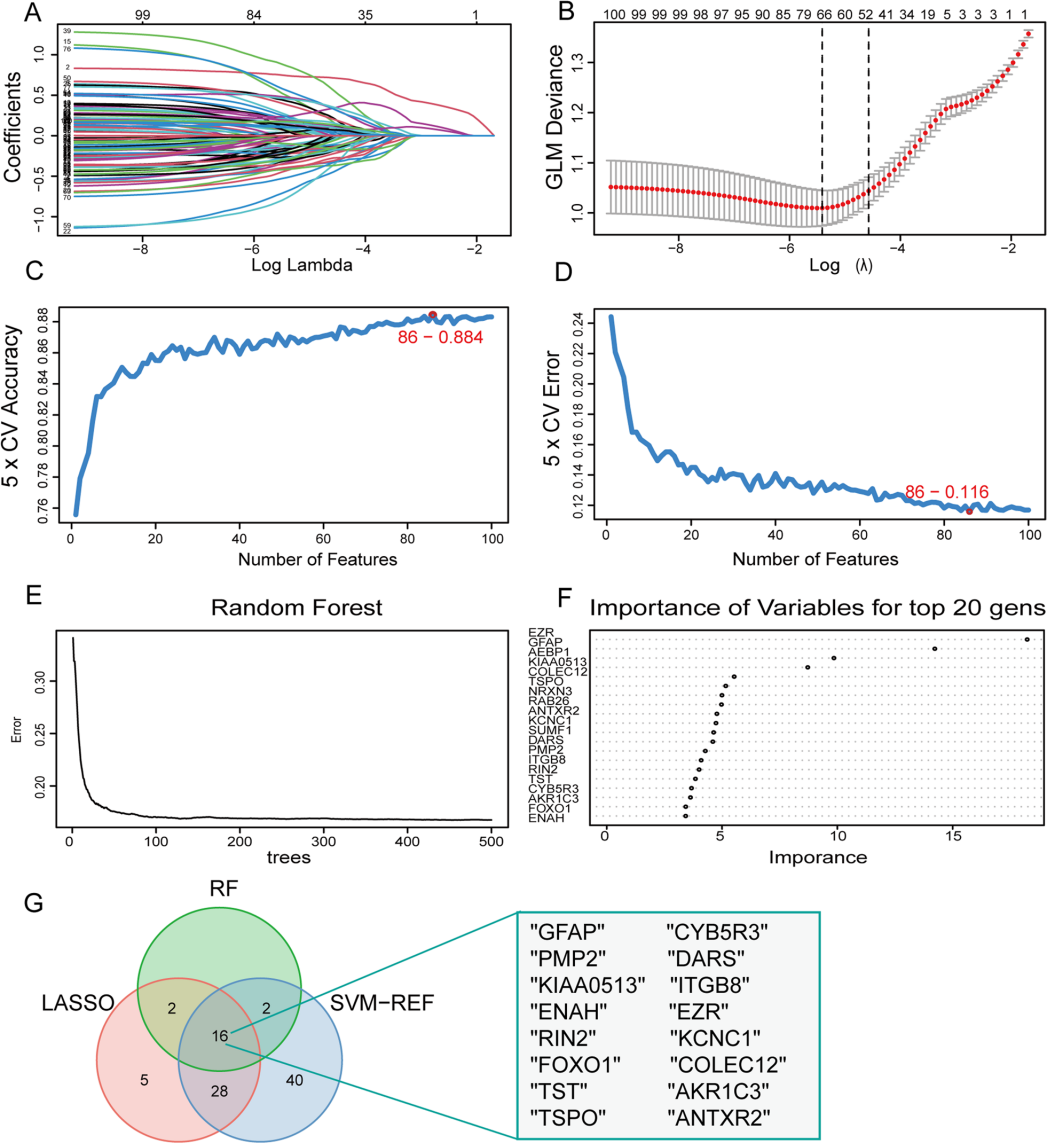
Screen for potential AD biomarkers. **A**, **B** Genes identified by the LASSO algorithm. **C**, **D** Optimal biomarker selection using the SVM algorithm. **E**, **F** The top 20 genes selected by the RF algorithm. **G** Venn diagram showing the diagnostic markers intersected by LASSO, SVM, and RF algorithm

We conducted three different machine-learning algorithms to screen for potential AD biomarkers. Using the LASSO regression algorithm, the hub genes were narrowed down to 51 variables (Fig. 7A, B). Using the SVM-REF algorithm, we identified a subset of 86 features among the hub genes (Fig. 7C, D). The RF algorithm revealed the top 20 feature genes (Fig. 7E, F). The overlapping genes among the LASSO, RF, and SVM-REF algorithms (*GFAP*, *CYB5R3*, *PMP2*, *DARS*, *KIAA0513*, *ITGB8*, *ENAH*, *EZR*, *RIN2*, *KCNC1*, *FOXO1*, *COLEC12*, *TST*, *AKR1C3*, *TSPO*, and *ANTXR2*) were selected for further study (Fig. 7G). Finally, we used logistic regression to out 8 feature genes (*GFAP*, *CYB5R3*, *DARS*, *KIAA0513*, *EZR*, *KCNC1*, *COLEC12*, and *TST*; p < 0.05) from the above 16 overlapping genes.

### Development and validation of the feature genes diagnostic signature for AD

**Fig. 8:**
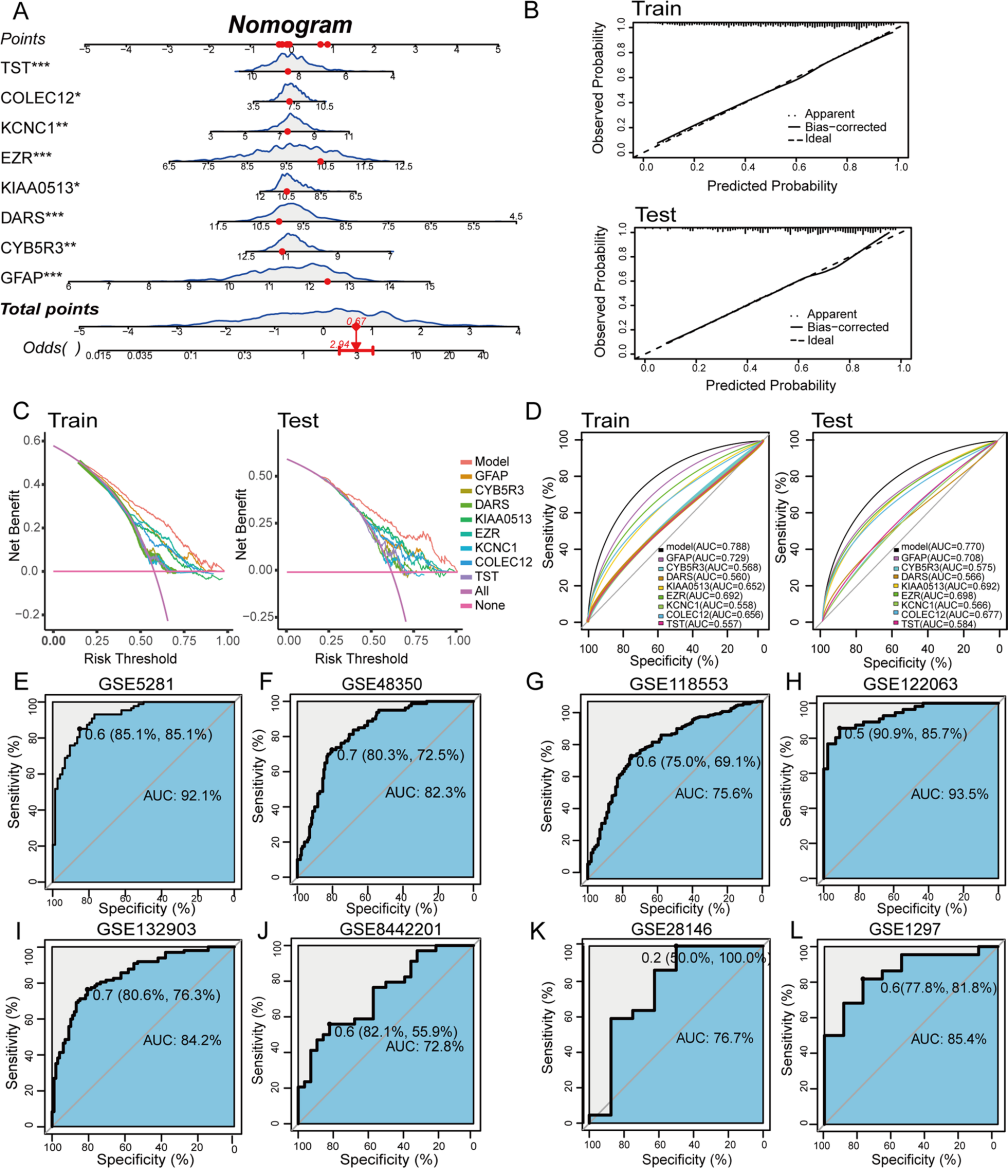
Validation of the diagnostic efficacy of the feature genes. **A** Nomogram displaying the predicted risk for AD based on the feature genes. **B** Calibration curve showing the predicted performance of the nomogram in internal datasets including the training and testing datasets. **C** DCA showing the clinical benefits of the nomogram in the internal datasets, including the training and testing datasets. **D** ROC curves showing the diagnostic performance of the feature genes in the internal datasets, including the training and testing datasets. **E**–**L** ROC curves showing the diagnostic performance of genes identified in the eight datasets, including GSE5281 **E**, GSE48350 **F**, GSE118553 **G**, GSE122063 **H**, GSE132903 **I**, GSE8442201 **J**, GSE28146 **K**, and GSE1297 **L**. (ns indicates no significance, *P < 0.05, **P < 0.01, ***P < 0.005, ****P < 0.0001)

A nomogram model was developed for AD diagnosis utilizing the eight feature genes (*GFAP*, *CYB5R3*, *DARS*, *KIAA0513*, *EZR*, *KCNC1*, *COLEC12*, and *TST*) (Fig. 8A). A calibration curve was used to assess the predictive capabilities of the nomogram model in the training and testing datasets. The calibration curve revealed a small error between the actual and predicted risk for AD, suggesting a high accuracy of the nomogram model for predicting AD (Fig. 8B). DCA revealed that the “nomogram” curve was higher than the curves representing “intervention for none,” “intervention for all,” and all single genes, suggesting that the patients may benefit from the nomogram model at a high-risk threshold from 0 to 1, and the clinical benefit of the nomogram model was higher compared with that of the single gene curve (Fig. 8C). Subsequently, the receiver operating characteristic (ROC) curve analysis was employed to evaluate the diagnostic capability of each feature gene for predicting AD progression in the internal datasets. The AUC values in the training dataset were 0.788 for the nomogram model, 0.729 for GFAP, 0.692 for EZR, 0.656 for COLEC12, 0.652 for KIAA0513, 0.698 for CYB5R3, 0.560 for DARS, 0.558 for KCNC1, and 0.557 for TST (Fig. 8D). The AUC values for the ROC curves in the testing set were 0.770 for nomogram model, 0.708 for GFAP, 0.698 for EZR, 0.677 for COLEC12, 0.692 for KIAA0513, 0.575 for CYB5R3, 0.566 for DARS, 0.566 for KCNC1, and 0.584 for TST (Fig. 8D). In addition, eight single validation datasets (GSE5281, GSE48350, GSE118553, GSE28146, GSE122063, GSE132903, GSE8442201, GSE28146, and GSE1297) were used to further confirm the diagnostic efficacy of these eight feature genes (Fig. 8E–L). To some extent, these results also suggest that the eight genes have a significant role in AD pathogenesis.

### Validation of the feature genes expression

**Fig. 9:**
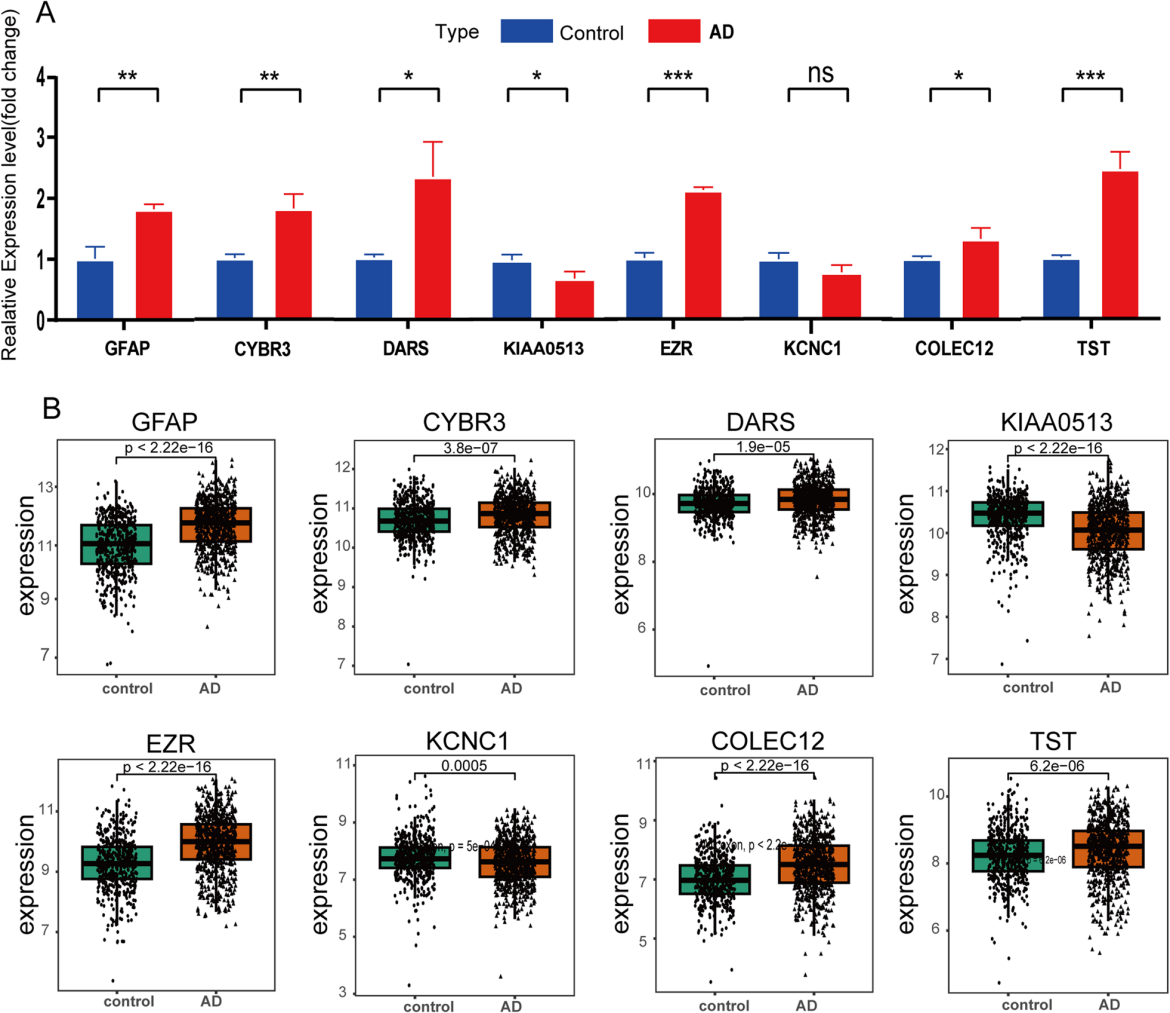
Validation of the eight feature genes expression between control and AD groups in mice **A** and the GEO datasets **B**. **A** The relative expression of eight genes was measured via qRT-PCR using mouse tissues (n = 3 in the control mice; n = 3 in the AD mice). **B** Expression levels of the eight genes in the GEO datasets. (ns indicates no significance, *P < 0.05, **P < 0.01, ***P < 0.005, ****P < 0.0001)

The differential expressions of the feature genes were verified in the aforementioned combined dataset (including GSE48350, GSE5281, GSE28146, GSE122063, GSE118553, GSE8442201, GSE132903, and GSE106241), which further demonstrated their diagnostic capacity for AD (Fig. 9B). In addition to the dataset, we further verified the expression of these eight feature genes by qRT-PCR analysis using tissues collected from AD mice or controls. Consistent with the bioinformatics analysis results, the expression of *GFAP*, *CYB5R3*, *DARS*, *EZR*, *COLEC12*, and *TST* were significantly higher in AD mice compared with controls, whereas *KIAA0513* exhibited significant downregulation (Fig. 9A). In contrast, *KCNC1* expression was not statistically different between the AD and control groups.

## Discussion

AD is a neurodegenerative disease wherein Aβ and NFT aggregation causes the loss of synapses, neuronal death, and subsequent memory impairment. There is a large heterogeneity in AD pathogenesis among patients, and thus, AD progression biomarkers need to be further refined [27, 28]. Accordingly, suitable AD subtypes and more powerful biomarkers are necessary for improved diagnosis and therapy.

Accumulating evidence suggested that the occurrence and progression of AD is closely related to substance and energy metabolism. Glucose, lipids, and energy metabolism has an important impact on AD [29–31]. The energy of the brain is primarily dependent upon glucose, which is metabolized to ATP via glycolysis, tricarboxylic acid (TCA) cycle, and electron transport chain [32]. Glucose metabolism is markedly decreased in the AD brain. Attenuated ATP production due to inefficient glucose utilization is accompanied by signal transduction breakdown, ionic pump dysfunction, and neurotransmission failure, ultimately leading to neuronal degeneration and death [29]. Lipids are also involved in AD pathology [33]. Apolipoprotein E ε4 (APOE4) is the strongest genetic risk factor for AD and drives metabolic dysregulation in astrocytes and microglia, leading to cholesterol accumulation, decreased neuronal excitability, and neuroinflammation [34, 35]. Restoring metabolic homeostasis can exert a significant neuroprotective effect [36]. Despite evidence implicating disrupted metabolism as pathological mechanism underlying AD, the precise genes and biological functions are yet to be identified, particularly the role of metabolism in regulating AD immunity.

In this study, to identify AD subclasses associated with metabolic processes, an AD classification was built based on metabolic genes from previous publications. Three distinct AD subclasses (MCA, MCB, and MCC) were identified. We explored the clinical features, metabolic signatures, and immune infiltration profile of each subclass. The results indicated that MCA exhibited specific metabolic signatures and was accompanied by high AD progression signatures (β-secretase activity, γ-secretase activity, NFT, Braak, and the AD-risk gene APOE4).

MCA was primarily associated with carbohydrate and lipid metabolism genes. The carbohydrate metabolism in MCA primarily involves glycolysis, fructose, mannose, and galactose metabolism, whereas the citrate cycle and pyruvate metabolism were decreased compared with the other two subclasses, indicating a reduction in the TCA cycle and glucose utilization (thereby reducing ATP production). Meanwhile, lipid metabolism in MCA mainly involves fatty acid degradation, probably due to low ATP production, which prompts a shift in energy metabolism to the ketogenic pathway. These metabolic disorders affect the energy supply of neurons in the brain. Furthermore, previous studies confirmed that mitochondrial ATP-synthase α subunit is lipoxidized and ATP-synthase activity was obviously reduced in the entorhinal cortex of patients with AD compared with the controls [37]. An analysis of the clinical features and metabolic signatures revealed that high APOE4 expression, NFT accumulation, and significant metabolic disorders were observed in the MCA subclass, thus presenting a poorer prognosis. Immune infiltration analysis suggested that MCA had an augmented immune score and a relatively higher abundance of immune cell infiltration compared with MCB and MCC. A significant change in the immune cell ratio was observed in the AD subclasses in which MCA exhibited higher levels of regulatory T cells (Tregs), CD4 + T cells, memory CD4 + T cells, B cells, activated dendritic cells, macrophages, and neutrophils compared with MCB and MCC, consistent with the findings of previous studies [38–40]. In addition, MCA exhibited a high stromal score and infiltration with endothelial cells and fibroblasts. Immune checkpoint genes that represent the potential targets for immunotherapy, such as *CD274* (PDL1) and *PDCD1* (PDL2), were primarily increased in the MCB.

To further elucidate the genomics characteristics of the AD subclasses, we used a combined dataset to the construct coexpression networks via WGCNA. The cyan module was positively correlated with MCA and the “A/T/N” system, such as NFTs, further supporting our hypothesis that the MCA subclass is a high-risk subclass for AD. Functional enrichment analysis revealed that the hub genes in the cyan module were primarily enriched in cellular morphological regulation and synapse-related functions and pathways. The impaired TCA cycle in the MCA is the main function of the mitochondria. These metabolic disorders may lead to mitochondrial dysfunction, inadequate energy supply, and massive reactive oxygen species release, inducing oxidative stress and calcium regulation imbalance, ultimately triggering neuronal apoptosis and synaptic loss [8].

Recently, various machine-learning algorithms have been used to identify new biomarkers and offer insights into disease pathogenesis, owing to an outstanding performance in diagnosis [41, 42]. Therefore, we used three machine-learning algorithms to further narrow down the number of hub genes. Eight feature genes were finally identified, including *GFAP*, *CYB5R3*, *DARS*, *KIAA0513*, *EZR*, *KCNC1*, *COLEC12*, and *TST*. *GFAP* is an astrogliosis marker. Recently, Shen et al. reported that plasma *GFAP* is significantly elevated from the preclinical stage of AD and is a promising diagnostic and predictive biomarker that distinguishes AD from the controls and non-AD dementia [43]. *CYB5R3* encodes cytochrome b5 reductase 3, which is essential for reductive reactions, such as cholesterol biosynthesis, fatty acid elongation, methemoglobin reduction, and drug metabolism [44]. *CYB5R3* expression was elevated in the human cortex in an AD proteomics study [45]. As an aspartyl-tRNA synthetase, *DARS* missense mutations caused a significant pattern of hypomyelination, motor abnormalities, and cognitive impairment [46]. A bioinformatics analysis suggested that *KIAA0513* reduction serves as a potential biomarker for early AD diagnosis [47]. *EZR*, which is a member of the ezrin–radixin–moesin protein family, has been recognized as a regulator of the adhesion signal pathways. *EZR* plays a key role in promoting the invasion and metastasis of malignant tumors [48]. *KCNC1* encodes a subunit of the Kv3 voltage–gated potassium channels and is associated with various human diseases, including ataxia, epilepsy, and developmental delay [49]. *COLEC12* encodes a member of the C-lectin family, which is a scavenger receptor that plays a crucial role in the binding and clearance of Aβ [50]. *TST* is an enzyme that is widely distributed in both prokaryotes and eukaryotes, which plays a crucial role in mitochondrial function [51]. These along with our findings are concordant and indicate that the overexpression of *GFAP*, *CYB5R3*, *DARS*, *EZR*, *COLEC12*, and *TST* as well as the downregulation of *KIAA0513* and *KCNC1* can predict poor AD prognosis. In addition, the nomogram model, calibration curves, DCA, and ROC curves verified the satisfactory diagnostic ability of these eight feature genes.

To the best of our knowledge, this was the first study to classify ADs from the perspective of metabolism. The screening and validation of the feature genes provided potential molecular targets for further exploring the metabolic mechanism of AD. However, this study had some limitations. First, the feature genes were only validated in AD mice and supporting human samples were lacking. Second, *KCNC1* showed inconsistent results in the AD datasets and AD mice, possibly due to the small mice sample size. Finally, the mechanism underlying metabolism regulation in AD warrants further investigated in vitro and in vivo, which will be our focus in future studies.

## Conclusion

We found a strong relationship between the metabolic status and AD pathogenesis using a comprehensive bioinformatics analysis. Three AD subclasses from the perspective of metabolism were identified with substantial differences in clinical characteristics, metabolism signatures, and immune infiltration. The results can better elucidate the heterogeneity of patients with AD. In addition, we identified and verified eight feature genes, *GFAP*, *CYB5R3*, *DARS*, *EZR*, *COLEC12*, and *TST*, which showed high expression, whereas *KIAA0513* and *KCNC1* displayed showed low expression in AD. The diagnostic model built by these eight genes exhibited outstanding diagnostic value. These findings provide a basis for more accurate and early AD diagnosis.

### Supplementary Information

**Additional file 1: Figure S1.** Consensus clustering matrix for k = 2–5.

**Additional file 2: Figure S2.** Box-plot of tissue original of AD subclasses.

**Additional file 3: Table S1.** GEO datasets information. **Table S2.** Primer sequences of feature genes. **Table S3.** KEGG pathway enrichment analyses of hub genes in cyan module. **Table S4.** GO enrichment analyses of hub genes in cyan module.

## Data Availability

The datasets analysed during the current study are available in the GEO database (https:// www. ncbi. nlm. nih. gov/ geo/), openly available for free download.

## Abbreviations

AD: Alzheimer's disease
Aβ: Amyloid-β
NFT: Neurofibrillary tangles
WGCNA: Weighted correlation network analysis
SVM: Support vector machines
RF: Random forest
GEO: Gene expression omnibus
GSVA: Gene set variation analysis
GO: Gene ontology
KEGG: Kyoto encyclopedia of genes and genomes
AUC: Area under the curve
qRT-PCR: Quantitative reverse-transcription polymerase chain reaction
PCA: Principal component analysis
DCA: Decision curve analysis
TCA: Tricarboxylic acid
APOE4: Apolipoprotein E ε4
