# Supplementary figures and images for: Identification of metabolism-related subtypes and feature genes in Alzheimer’s disease
Source: J Transl Med. 2023 Sep 15;21:628. doi: 10.1186/s12967-023-04324-y (PMC10504766; doi:10.1186/s12967-023-04324-y)

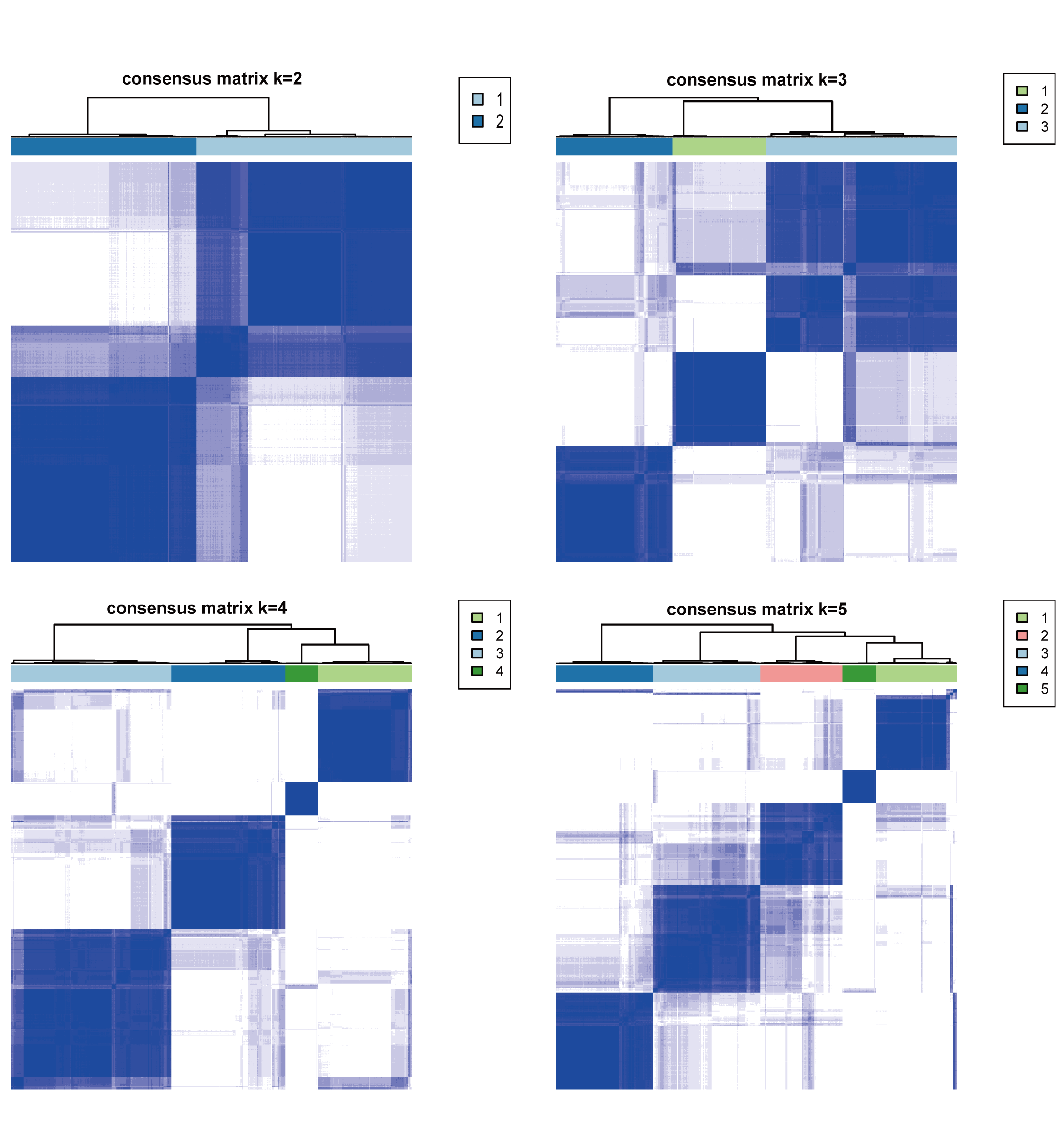

Supplement: Supplementary file 1 — Additional file 1: Figure S1. Consensus clustering matrix for k = 2–5. [file 12967_2023_4324_MOESM1_ESM.tif]

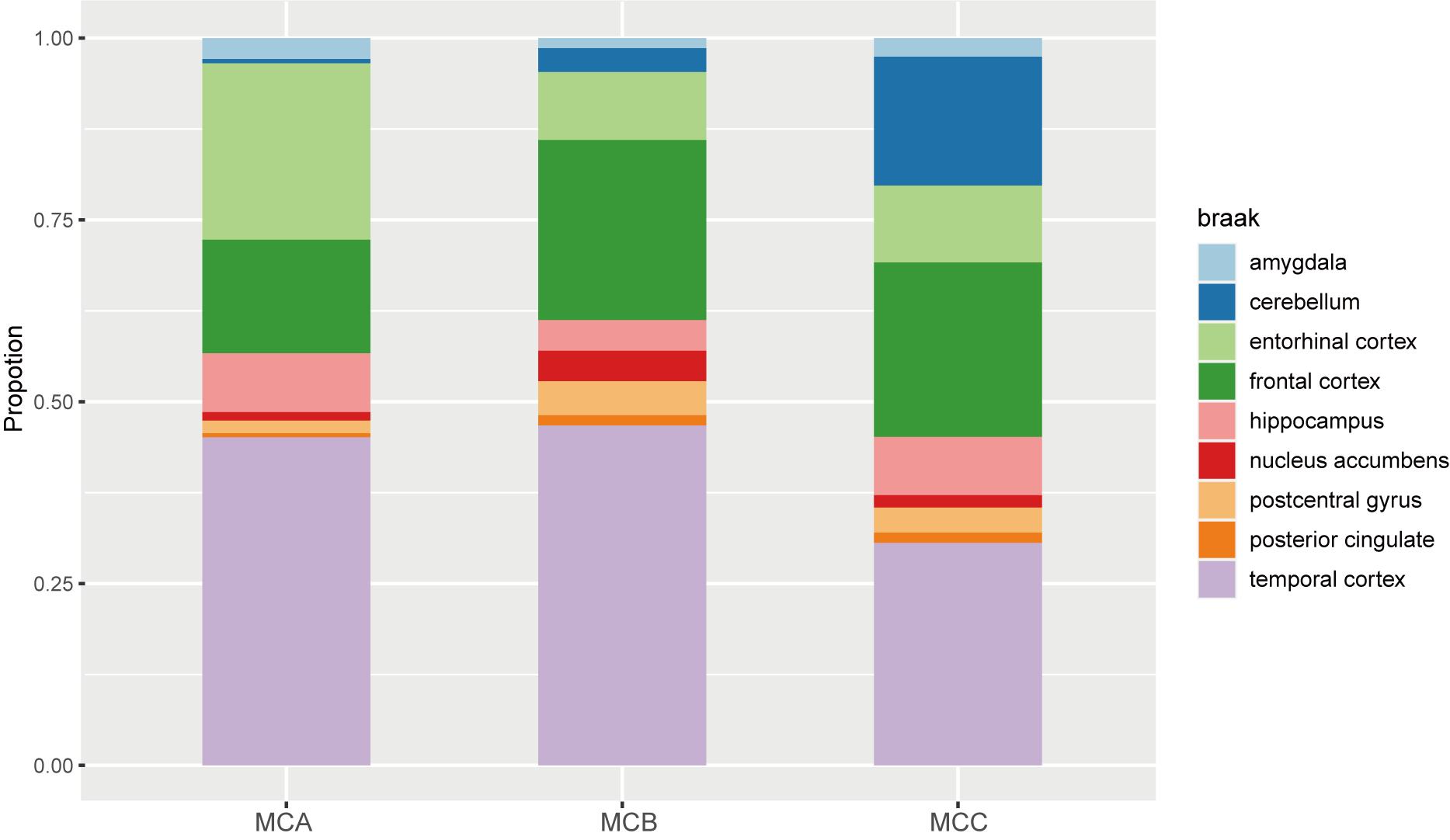

Supplement: Supplementary file 2 — Additional file 2: Figure S2. Box-plot of tissue original of AD subclasses. [file 12967_2023_4324_MOESM2_ESM.tif]
